# Long-read sequencing and benchmarking of saliva against blood for genomic variant detection in autism

**DOI:** 10.64898/2026.10.09.26365004

**Published:** 2026-10-13

**Authors:** Yang Sui, Gabriele Campos, Youngjun Kwon, Isaac Wong, Nidhi Koundinya, Katherine M. Munson, Kendra Hoekzema, Marcelo Ayllon, Tianyun Wang, Emily Neuhaus, Evan E. Eichler

## Abstract

Autism is highly heritable, yet many cases remain genetically unsolved even after short-read sequencing (SRS). Long-read sequencing (LRS) increases sensitivity for pathogenic variant discovery, especially for complex structural variants (SVs) in repetitive regions missed by SRS. However, LRS largely relies on blood-derived DNA; whether saliva can support genome-wide LRS at population scale remains to be seen. We report the first systematic evaluation of saliva-derived DNA for genome-wide LRS in autism, benchmarking 105 new saliva samples against 68 new blood samples and integrating 189 previously published LRS blood samples to expand the autism cohort and improve ultra-rare pathogenic variant discovery. A matched blood-saliva quad provided direct comparisons of variant detection between sample sources. Saliva samples contained substantially more nonhuman DNA than blood (mean 10% versus 0.005% of reads), reducing mean effective genome coverage from an estimated 28X to 24X after removal of nonhuman reads, compared with 34X in blood samples. Despite this reduction, saliva and blood samples yielded comparable numbers of validated (22,591 versus 23,064) and rare (209 versus 213) SVs per sample, which was also observed in the quad when matching coverage. Saliva LRS identified four new pathogenic and likely pathogenic variants missed by SRS. We report variants affecting *MECP2* , *DNMT3A* , *CHD8* , *LMBRD2* , *POGZ* , and *PHF3* ; four additional candidates with potential relevance to autism; and an increase of diagnostic yield of 9-18%. Saliva-derived DNA support blood-comparable discovery of pathogenic variants missed by SRS, including SVs, with increased sequencing coverage compensating for nonhuman DNA contamination and reduced assembly contiguity, thus showing saliva DNA collection as a feasible strategy for large-scale LRS studies of patients and population-scale sequencing of cohorts.

